# Redefinition of Successful Treatment of Patients With Hypothyroidism. Is TSH the Best Biomarker of Euthyroidism?

**DOI:** 10.3389/fendo.2022.920854

**Published:** 2022-06-16

**Authors:** Stephen P. Fitzgerald, Henrik Falhammar

**Affiliations:** ^1^ The Departments of General Medicine and Endocrinology, The Royal Adelaide Hospital, Adelaide, SA, Australia; ^2^ School of Medicine, The University of Adelaide, Adelaide, SA, Australia; ^3^ Department of Endocrinology, Karolinska University Hospital, Stockholm, Sweden; ^4^ Department of Molecular Medicine and Surgery, Karolinska Institutet, Stockholm, Sweden; ^5^ Menzies School of Health Research and Royal Darwin Hospital, Tiwi, NT, Australia

**Keywords:** physiology, euthyroidism, thyroid function, thyrotropin (TSH), thyroid hormones

## Abstract

In recent years evidence has accumulated supporting a revised view of the nature of euthyroidism and the biomarkers of thyroid function. Within the normal range, variations in thyroid hormone levels are associated with variations in clinical parameters and outcomes. There are therefore no readily identified individually specific optimum levels of thyroid hormones for any individual. Levels around the middle of the normal population range may best reflect euthyroidism. These levels may have evolutionary advantages on the basis that adverse outcomes often increase with divergence from such levels, and physiological processes tend to minimise such inter-individual and intra-individual divergence. In populations of predominantly untreated individuals, levels of thyroid hormones and in particular levels of free thyroxine (FT4) correlate more often with clinical parameters than do levels of thyrotropin (TSH). Levels of thyroid hormones may therefore be regarded as the best available biomarkers of euthyroidism and dysthyroidism. It follows that ‘subclinical hypothyroidism’ (normal FT4/raised TSH levels), rather than being an accurate marker of peripheral tissue hypothyroidism is more a marker of decreased thyroid reserve and prognosis. The recent evidence suggests that treatment of hypothyroxinemia, regardless of the TSH level, and monitoring therapy using FT4 and/or triiodothyronine levels, depending on the replacement regime, may result in more successful treatment of hypothyroidism than relying on thyrotropin levels for patient selection and subsequent treatment monitoring. The equivalents of mid-range levels of thyroid hormones (especially FT4), adjusted by individual comorbidity concerns, may be rational general replacement targets. These implications of the new evidence may create opportunities for novel trials of thyroid replacement therapy.

## Introduction

Since publication of the last American Thyroid Association guidelines (1) regarding hypothyroidism in 2014 there have been advances in the understanding of the fundamental aspects of thyroid action and regulation enabling reconsideration of the entity of euthyroidism in terms of its nature and biomarkers. In turn this has relevance to the diagnosis and management of patients with hypothyroidism.

Thyroid hormone is essential for normal development, growth, neural differentiation, and metabolic regulation in mammals (2). Thyroid hormone regulates a wide range of genes after its activation from thyroxine to triiodothyronine (2). At the cellular level the signalling pathway is complex and highly regulated due to the expression of cell and tissue-specific thyroid hormone transporters, multiple thyroid hormone receptor isoforms and interactions with corepressors and coactivators (2). In addition, non-genomic actions of thyroid hormone have been recognised (2).

The control of thyroid function and thyroid hormone levels is principally effected by the classical pituitary- thyroid feedback loop and the hypothalamic- thyroid feedback loop, these loops constituting the hypothalamic-pituitary-thyroid axis (3). There are additional feedback loops and other mechanisms contributing to thyroid hormone control and homeostasis (3).

Thyroid function tests (TFTs) include levels of thyrotropin (TSH), free thyroxine (FT4) and free triiodothyronine (FT3) (4). TFTs indicating hypothyroidism include elevated levels of TSH (unless the hypothyroidism is secondary) (4), low levels of FT4, and, if the hypothyroidism is more severe, low levels of FT3 (4–6). Tests indicating hyperthyroidism include low levels of TSH (unless the hyperthyroidism is secondary), and elevated levels of FT4 and/or FT3 levels (4).

Euthyroidism has previously been understood as being represented by a range of TFTs narrower than the population normal range, specific to each individual, set genetically, and that within this range each individual has an optimal exposure to thyroid hormones (1, 3, 7, 8). Despite some discrepancies (1) and objections (9), TSH levels have been regarded as the most sensitive of the TFTs (1, 2, 10, 11). Consequently, TSH levels have been promoted as the first and perhaps only necessary test for euthyroidism (11, 12), i.e., with some exceptions the understanding has been that a normal TSH implies euthyroidism and an abnormal TSH implies dysthyroidism. In the context of an abnormal TSH level subsequent measurement of the thyroid hormone levels has allowed classification of the dysthyroidism (4). The combination of normal thyroid hormone levels with abnormal TSH levels has been regarded as indicative of subclinical thyroid dysfunction (4).

Analogously, the management of patients’ replacement therapy for hypothyroidism (except for the small subset of secondary hypothyroidism) has been directed to target TSH levels (1, 11, 13, 14).

This paradigm has continued despite there being no empiric evidence that TSH levels are in fact the best guide to the thyroid state of peripheral tissues (as distinct from the thyroid state of the pituitary), or the best guide to successful replacement therapy of hypothyroidism. The pre-eminence of TSH levels has been a ‘given’, supported mostly by theoretical argument.

This review concerns these fundamental concepts in thyroidology. Different schools of thought and controversies are covered, and research gaps and potential developments are identified. In particular, we review the evidence indicating that previous concepts regarding euthyroidism, and the emphasis on TSH levels may no longer be sustained. We describe the evidence indicating that there are no such entities as ‘individual euthyroidism’ or thyroid set points and that euthyroidism is a more subtle concept whereby all levels of thyroid hormones are associated with different risk profiles such that no levels are readily identified as optimal for any individual. We also describe the evidence indicating that TSH levels may not be the best guide to the thyroid state and thereby not the best biomarkers of euthyroidism, being inferior in this regard to thyroid hormone levels and in particular to FT4 levels. We describe the inconsistencies in the theoretical rationale used to support the use of TSH levels for the assessment of the thyroid state. Finally, we indicate the relevance of all this evidence to, the definition of successful clinical diagnosis and management of patients with hypothyroidism, and the design of future clinical trials.

## Euthyroidism

The traditional view of euthyroidism is that each healthy individual’s normal TFTs represent a state of euthyroidism for that individual-i.e., a state whereby organs and tissues are exposed to optimal levels of thyroid hormones (1, 3, 8, 9). Implicit in this concept is that all organs and tissues of an individual tend to have similar requirements and that interindividual variation in the levels of TFTs indicating euthyroidism reflects variation in sensitivity to thyroid hormones.

However, multiple studies indicate that TFT variation within the normal range is associated with a variety of physiological and clinical parameters (15, 16). Well studied examples of outcomes associated with high normal TFTs (high normal thyroid hormone levels/low normal TSH levels) include atrial fibrillation (17, 18), low bone density (19, 20), cancer (21, 22), dementia (23) adverse pregnancy outcomes (24) and death (25, 26). Examples of outcomes associated with low normal TFTs (low normal thyroid hormone levels/high normal TSH levels) include fatty liver (27, 28), metabolic syndrome (29, 30) diabetes (31, 32), BMI (33, 34) and again, adverse pregnancy outcomes (35, 36). In some circumstances the relationship may be U-shaped (37). It may be that the associations with high normal TFTs are stronger or more likely to be causal whereas the associations with lower levels of TFTs may be more affected by reverse causation (38) or the effect of a more fundamental entity resulting in thyroid dysfunction and the relevant clinical parameter (39).

Supporting a causal link of the associations is the fact that the direction of these risks tend to follow the direction of risk associated with overt thyroid dysfunction. For example, the increase in risk of atrial fibrillation with increasing TFTs within the normal range reflects the well-recognised association of overt hyperthyroidism with atrial fibrillation. Also supporting a causal link is that some outcomes (for both low- and high-normal thyroid function) have been studied prospectively, the thyroid state pre-dating the appearance of the relevant clinical parameter (23, 25, 26, 32, 35, 36).

These associations of clinical outcomes with normal range function occur in individuals who are otherwise asymptomatic and therefore TFTs might be regarded as risk factors akin to cardiovascular risk factors. However, in the case of any variation in thyroid function some risks increase as others decrease.

The magnitude of the risks associated with variation within the normal range of thyroid function is not necessarily small (40). It can approximate and in some circumstances even exceed traditional risk factors (18, 21).

The gradation of the various risks within the normal range is thus evidence that the inter-individual variation in TFT’s does not represent a form of compensation whereby thyroid hormones are adjusted physiologically to suit the particular tissue sensitivity of each individual. Further evidence for this is found in the population correlations between FT4 and TSH levels. If individuals with higher levels of FT4 required such levels to be euthyroid on account of a relative peripheral tissue insensitivity to FT4 these higher levels of FT4 would need to be driven by some type of set point physiology whereby the thyroid state of the peripheral tissues (or at least of the hypothalamus-pituitary) was monitored (41), or alternatively, by matching central resistance to thyroid hormone resulting in higher TSH levels. In the presence of set point physiology the population FT4/TSH correlation would be positive (42). Similarly with central and peripheral thyroid hormone resistance relatively high FT4 levels would be accompanied by relatively high TSH levels again resulting in a positive correlation between FT4 and TSH in the population. These putative mechanisms are however denied by the empiric correlation which is in fact strongly negative across all ranges of thyroid function (43, 44). This negative relationship indicates that individuals with high-normal levels of FT4 tend to have thyroid glands relatively sensitive to TSH stimulation rather than tissues relatively insensitive to FT4.

It follows from this gradation of risks across the normal range that rather than there being a dichotomy of euthyroid and dysthyroid levels of TFTs, there is a continuum of chance of thyroid hormone related outcomes associated with variation in TFTs (40). Any individual’s particular TFTs are associated with a particular risk profile of a number of outcomes and any change to these levels results in some risks increasing and others decreasing.

It is therefore difficult to define levels of TFTs that are truly ideal or best possible for any individual. And even if there were at a point in time levels that on balance offered the best chances of ongoing health for any individual, any change in non-thyroid related pathophysiology might then render these levels disadvantageous.

This conception of the variation in risk associated with variation within the normal range is not unique to thyroid physiology with similar concepts applying to adrenal and sex hormone physiology (45, 46).

Given that all levels of thyroid function are associated with risks and benefits, it follows that there is in particular no need to consider that any hypothyroid individual’s usual pre-morbid thyroid hormone levels were necessarily especially euthyroid.

On the other hand, physiological processes such as TSH autoregulation (47), and FT4/TSH feedback regulation (48) act to minimise inter-individual and intra-individual variation in TFTs, such that these levels cluster around the middle of the population range (49). This would suggest that there are evolutionary advantages to such levels. Furthermore, risks of adverse outcomes and the likelihood of symptoms tend to increase as TFTs increasingly diverge from mid-range levels (38). Thus, the normal population range of TFTs, and particularly mid-range levels, can reasonably be considered to reflect euthyroidism. Levels at the edges of the normal range might be considered to reflect borderline thyroid function. The indication for replacement therapy therefore increases the more TFTs diverge from midrange levels.

## The Best Biomarker of Euthyroidism Is FT4

The assessment of thyroid function and thereby the definition of euthyroidism relies on biochemical thyroid function testing (1, 11, 12). Possible alternative biomarkers to assess the supply of the organism with thyroid hormones include a variety of physiological variables sensitive to thyroid hormones, such as resting heart rate and other determinants of cardiac output (3), Achilles’ tendon relaxation time (50), plasma levels of lipids (50) and other parameters. However, tissue biomarkers of thyroid hormone action are not recommended for routine clinical use, outside of the research setting, since these parameters are not sensitive, specific, readily available, or standardized (1, 3, 50). Similarly, patients’ symptoms, whether physical or psychological, though of some value (51) lack sensitivity and specificity (1, 50).

On account of the relative variations in, and/or lack of independence between, the curves describing the TSH responses to FT4 and T3, and the curves describing the FT4 and T3 responses to TSH (52) there is a strong, negative population correlation in the population between FT4 and TSH (43, 44), and between T3 and TSH (53). Therefore, any relationships between TSH and clinical parameters tend to be similar to those between FT4 or FT3 and clinical parameters but in the opposite direction. Nevertheless, recent work has now provided evidence indicating how FT4, FT3 and TSH compare in the assessment of the thyroid state of peripheral tissues and thereby how they compare as markers of euthyroidism.

We have performed a meta-analysis of studies recording associations of clinical parameters with TSH, FT4 and T3/FT3 levels taken simultaneously (38). T3 or FT3 had been analysed in different studies but the results were analysed together in this meta-analysis as there were relatively few studies of each. Clinical parameters included those related to low bone density, cardiac disease, cancer, dementia, frailty, metabolic syndrome, mortality, and pregnancy. Overall, there were overwhelmingly more associations of clinical parameters with FT4 and FT3/T3 levels than with TSH levels (p< 0.0001). Despite the historical preference for TSH levels no clinical parameters associated better with TSH levels than with FT4 and T3/FT3 levels. The superior associations with FT4 in particular were universal when the data was analysed according to clinical parameter, size of study and number of covariates. The associations with FT4 were more robust than those of T3/FT3 and persisted when the data was analysed for possible reverse causation.

Other meta-analyses restricted to single clinical outcomes (17, 54, 55) have shown similar results, as have subsequent studies (56). Previous authors had commented on the apparent superiority of thyroid hormones, but some had believed their results may have been discrepant or isolated to their studied parameter (30, 34). It has however previously been suggested that TSH may be more a marker of the pituitary status than that of peripheral tissues (40).

Given that the clinical thyroid state is a sum of clinical parameters, and that in the above studies, no clinical parameter associated better with TSH levels than thyroid hormone levels, and that one of the examined parameters was mortality, there is strong evidence against the proposition that TSH levels provide the best guide to the thyroid state of peripheral tissues. The evidence strongly suggests FT4 levels are the best single biomarkers of euthyroidism. The measurement of FT4 levels rather than TSH levels as well as being a better general test for euthyroidism also covers the possibility of secondary thyroid dysfunction that might be missed with screening TSH testing (57).

Despite the evidence showing that FT4 is the superior biomarker of euthyroidism, it does not follow necessarily that in clinical practice we should merely switch from measuring TSH levels to another single test, that of FT4 levels. The measurement of both FT4 and FT3, or both FT4 and TSH provides more precision still, covering the possibility of T3 toxicosis. The measurement of TSH may provide information apart from the state of the peripheral tissues i.e., prognostic information (58), and an indication of the state of the thyroid gland itself. Cost- benefit studies might inform discussion as to guidelines in this regard.

It follows that ‘subclinical hypothyroidism’, a state of normal FT4 levels and raised TSH levels, should be more correctly be recognised as describing a spectrum of individuals from euthyroidism to borderline hypothyroidism, depending on the FT4 level. The elevation of the TSH levels signifies a healthy compensatory homeostatic response of the hypothalamus -pituitary mitigating any deficiency in thyroid gland function (3, 9), such that the peripheral tissues are protected to a greater or lesser extent. Without such a rise in TSH levels one would expect a lower level of thyroid hormones (9). Several studies have in fact found associations of parameters with low FT4 levels but not with subclinical hypothyroidism (36, 59–62).

The evidence indicates that borderline or subclinical hypothyroidism would more correctly be defined by levels of FT4 around the lower end of the range, regardless of the TSH levels. Isolated hypothyroxinemia would then be a subset of borderline hypothyroidism, possibly representing a mild non- progressive form of secondary hypothyroidism (3, 63). Because of the population correlations of FT4 and TSH levels there would be considerable overlap of individuals with traditionally defined subclinical hypothyroidism and individuals with borderline low FT4 levels.

In summary, in the section ‘Euthyroidism’ we presented the evidence that euthyroidism is a continuum and that dysthyroidism increases with variation away from the mid-range of thyroid function tests. In this section we presented the evidence indicating that of the thyroid function tests, most emphasis may be placed on FT4 levels. In terms of the management of hypothyroidism, there is strong evidence that potential patients too may be assessed on the basis of FT4 levels rather than TSH levels. ‘Subclinical hypothyroidism’ is not the most accurate classification of borderline hypothyroidism of peripheral tissues and organs.

## Euthyroidism in Patients on Replacement Therapy

Given that it is difficult, if at all possible, to be precise with defining/identifying ‘individual euthyroidism’ (3) the goal of precision for the restoration/definition of euthyroidism in the treatment of any individual with hypothyroidism may be unrealistic. The physiological and psychological symptoms of untreated hypothyroidism themselves lack sensitivity and specificity (64, 65), and attributing symptoms in borderline cases is problematic (66). The goal of restoring complete symptomatic health in patients with residual symptoms on replacement therapy despite normal range TFTs with subtle adjustments of replacement therapy in the hope of restoring ‘individual euthyroidism’ is therefore ambitious.

Rather than being associated with hormone levels appropriate for the individual, a feeling of well-being seems to be more associated simply with higher levels of thyroid hormones (67). This holds to the point of ‘subclinical hyperthyroidism’ (68) but overt hyperthyroidism results in decrements rather than further improvement (69). The improvement in well-being with higher levels of thyroid hormones has been associated with a slight impairment of physical health (68). Overall, the evidence supports attempts to improve well-being by at least lifting thyroid hormone levels to mid-range levels, rather than relying on reported wellbeing to assess optimal replacement therapy.

Though mid-range levels of thyroid hormones and in particular FT4 appear to be the best indicators of euthyroidism in the untreated state, care must be taken extrapolating this to the monitoring of replacement therapy. The studies that showed the superior association of FT4 with clinical parameters analysed populations that contained relatively few individuals on replacement therapy. There was no evidence that this sub-group behaved differently and though by extrapolation one would not expect this physiological property to change in the event of thyroid replacement therapies, further studies are required to confirm that in patients on replacement therapy FT4 and FT3 levels again perform better than TSH levels.

At this stage there is minimal evidence concerning the monitoring of the thyroid state with replacement therapy. There is evidence that monitoring TSH levels with the aim of keeping them within range provides superior outcomes as compared with outcomes in individuals with TSH levels outside of this range (70, 71). Psychological outcomes have been shown to be associated with FT4 and TSH levels in patients on T4 replacement therapy (72). However, we are not aware of any studies comparing a broad range of outcomes with the monitoring of thyroid hormone levels rather than TSH levels. This is of course not surprising as it has been assumed for decades that TSH levels are the best indicator of the thyroid state.

Other considerations include that replacement therapies may vary as to the hormones prescribed (1). Though replacement with levothyroxine is generally recommended alternative therapies include desiccated thyroid extract, liothyronine and combinations of levothyroxine and liothyronine (73). These different therapies result in different diurnal variation in hormone levels and FT4:FT3 ratios in the circulation; high ratios with levothyroxine therapy and low ratios with desiccated thyroid extract therapy (73). Patients’ replacement therapy may also be affected by different residual levels of thyroid gland function (1, 48). The physiology of the thyroid FT4/TSH feedback loop may be altered in replacement therapy and the anticipated tissue response to levels of FT4 too may need recalibration (9).

The general use of thyroid hormone and in particular FT4 levels as rational targets for replacement therapy for primary hypothyroidism would unify the targets with those of secondary hypothyroidism where already thyroid hormone levels are the recommended parameters to monitor (1, 57).

Regardless of any changes to the method of monitoring thyroid replacement therapy, TSH levels would remain useful for monitoring therapy in the context of thyroid neoplasm where a goal is to suppress TSH levels rather than restoring tissue euthyroidism only (74).

The almost universal finding that the treatment of mild hypothyroidism results in no significant benefit (75) stands as evidence against the usefulness of TSH levels in diagnosis and management in these circumstances. Most of the trials of the treatment of mild hypothyroidism have selected patients with sub-clinical hypothyroidism. As previously stated the evidence indicates that this selection criterion is flawed and many such chosen patients with levels of thyroid hormone just below the middle of the population range would have had minimal opportunity for improvement. Furthermore, because changes to TSH levels are so sensitive to changes in FT4 levels, replacement therapy to normalise TSH levels may have resulted in only minimal changes to the FT4 levels [2 pmol/L in the TRUST study (76)]. Treated individuals with low levels of FT4 may thus have remained mildly hypothyroid. Many of treated patients thus would either have not been in a position to benefit or would have received potentially inadequate therapy.

The evolving understanding of euthyroidism and its assessment provides the opportunity to repeat studies of the levothyroxine treatment of mild hypothyroidism, with selection and monitoring of patients on the basis of FT4 levels (77) regardless of TSH levels (78). Recommendations that the treatment of subclinical hypothyroidism be restricted to individuals with especially high levels of TSH (79) does in fact indirectly select patients with lower FT4 levels.

There have been few studies of treatment of hypothyroxinemia with thyroid replacement therapy. These appear to have been restricted to the study of cognitive outcomes in the offspring of pregnant women (80, 81). These studies have been negative, but they have been criticised in that they may have been underpowered, and therapy may have been started too late in pregnancy (1, 39).

Furthermore, the knowledge of the associations of clinical parameters with particular levels of thyroid hormones allows for the possible personalisation of mid-range replacement targets, i.e., any personalisation may be better effected *via* FT4 levels than the recommended TSH levels (1). For example, the associations of the risks of dementia, death, and atrial fibrillation with higher thyroid function, support the opinion that older individuals might be regarded as being ‘most euthyroid’ with FT4 values towards the lower end of the range (1, 77). Younger patients at risk of the metabolic syndrome might do better with high normal FT4 levels, pregnant women might be best to be midrange. Beyond these considerations there is also scope for patient choice in that patients might prefer a particular thyroid replacement regimen (82) and accept the associated risks that come with any benefits. Thus, patient characteristics may have relevance for the selection of patients for replacement therapy and the vigour of any such therapy (77).

Similar considerations for further research regarding replacement therapy apply to replacement therapies other than levothyroxine alone. In these circumstances the targets of therapy would logically correspond to the treatment regimen and include FT4 and/or FT3 levels as appropriate. It may be possible by calibration studies to define, for all forms of thyroid replacement, the hormone level equivalents of mid-range FT4 levels in untreated patients.

Though the evidence suggests that general TSH levels may not provide the most accurate targets for thyroid replacement therapy, it could be argued that once target hormone levels are initially attained in an individual, maintenance of the simultaneous TSH level in that individual presents a rational target for subsequent follow-up. In particular this might be an alternative to following the levels of both FT4 and FT3 in individuals treated with both levothyroxine and liothyronine.

Given the results thus far of the treatment of subclinical hypothyroidism, it remains possible that trials of the treatment of borderline hypothyroidism, however defined, and attempts at precision with thyroid replacement therapy in general, show minimal benefit. In these circumstances possible explanations might include that the lower and mid parts of population range of thyroid hormone levels are satisfactory and that the associations between borderline low thyroid hormone levels and clinical parameters represent reverse causation (83), result from other factors (39), or have been falsely identified/attributed (66). In these circumstances, treatment indications and thyroid hormone target levels might be reduced.

Separate studies would be required for the assessment of the value of treating mild hyperthyroidism as the associations of clinical parameters with high normal levels of thyroid hormones seem relatively more robust (38).

## Support for TSH Levels, FT4 and TSH Levels Combined, and T3 Levels as Biomarkers of Euthyroidism

The use of TSH levels to monitor the thyroid status are in accord with the 2014 guidelines (1) where it is stated that ‘TSH has been accepted as a robust, sensitive, and reproducible indicator of the thyroid status. Serum TSH is thought to integrate signals from both T4 and T3 and its dynamic relationship with these parameters has led to its establishment as the best single test for determining thyroid status. The logarithmic relationship between TSH and thyroid hormones bestows sensitivity: even if circulating T3 and T4 are in the normal range it cannot be assumed that the subject is euthyroid. The interindividual ranges for T3 and T4 are much broader than the individual variance, such that measuring T3 and T4 is a suboptimal way to assess thyroid status.’

This support for the use of TSH levels as the best biomarker for the euthyroid state has thereby been based on a theoretical physiological rationale and predates studies supplying the relevant empiric data.

Despite the publication of the data indicating the superiority of thyroid hormone levels over TSH levels there have been ongoing publications relying on TSH levels and the concept of subclinical hypothyroidism (84, 85). Some of these projects may well have commenced prior to the availability of this new data and thus relied on the dominant teachings of the time.

Otherwise however, concern has been expressed that though serum thyroxine has a better correlation with all-cause mortality and morbidity than serum TSH that this has limited relevance for the management of acquired hypothyroidism or thyrotoxicosis (13). Some of this concern relates to the fact that most of the relevant data was derived from individuals not on replacement therapy. Even in this situation however, it remains demonstrated that the diagnosis of thyroid disorders, and in particular mild thyroid dysfunction, is more accurately achieved by the measurement of FT4 than TSH.

This review also recommended continuation of the use of TSH assays to assess thyroid status because ‘it (TSH) is the most specific and sensitive index of the thyroid status’. We would argue that this is a statement both unsupported and denied by clinical data. Finally, it is recommended that the goal of treatment is ‘to restore the patient to his or her constitutive status….using serum TSH concentrations, as recommended by current societal guidelines’. As we have indicated, the available evidence does not indicate that an individual’s constitutive status is particularly euthyroid or that monitoring replacement therapy with TSH levels is superior to monitoring with levels of thyroid hormones.

Another commentary (14) has agreed that the use of a controlling hormone (TSH) to diagnose and differentiate thyroid disease is less than optimal but also proposed/repeated that in the context of thyroid replacement therapy ‘TSH gives an integrated answer (1) as to the adequacy of the replacement therapy’. In particular it was proposed that the TSH level compensates for variation in T3 levels. It was noted that the maintenance of normal TSH levels is associated with mortality comparable with background populations.

Certainly, at the moment further studies are required to compare TSH levels with thyroid hormone levels in the monitoring of thyroid replacement therapy. However, though the studies indicating the superiority of FT4 levels included relatively few patients on replacement therapy the results were similar with and without their inclusion. Given that TSH levels were previously regarded as the best indicators of the thyroid state in the untreated state it previously followed logically that TSH levels would be the best indicators of the adequacy of thyroid replacement therapy. It is another matter to claim that TSH levels are the best indicators of replacement therapy in the presence of evidence showing that thyroid hormone levels are superior indicators of the thyroid state in untreated individuals. With thyroid replacement, just as in the untreated state, any TSH level corresponds to a range of thyroid hormone levels in the population (43, 44). Finally, with optimal thyroid replacement, one might hope that the risk of death might be lower than that of a background population as such a population would include individuals with thyroid hormone levels that place them at an increased risk of death (56, 86).

The underlying rationale of euthyroidism, thyroid action and regulation used to support the primacy of TSH levels has included the concept of a ‘set point’ (1, 3, 16, 87). A set point model proposes that the body normally functions at levels of certain parameters that are optimal, that these levels are pre-set genetically, and that there are physiological mechanisms to monitor the levels and correct any perturbations (41). In the thyroid literature there are variations on this theme but in the end there has been general agreement that each individual has their own set point levels these levels indicating euthyroidism, i.e., there is a narrow range of thyroid hormone levels consistent with ‘individual euthyroidism’, which is more precise than, and to be distinguished from, euthyroidism as defined by thyroid hormone values lying within the population range (1, 3, 8).

Thus, there have been the concerns that individuals may have hypothyroidism even though the thyroid hormone and in particular TSH levels are apparently normal by virtue of such individuals having levels that had changed but still remained in the normal population range, i.e., their starting set-point was one with high normal thyroid hormone levels and/or low normal TSH levels (1, 8, 88).

Furthermore, as there is a log-linear (or similar) relationship between FT4 and TSH (43, 44, 88) small primary changes in FT4 levels result in larger changes in TSH levels. This relationship has been exploited to be regarded as a rationale to rely on TSH levels as sensitive indicators of variation from ‘individual euthyroid’ values to ‘individual dysthyroid’ values.

In the section ‘Euthyroidism’ we discussed the empiric evidence against ‘individual euthyroidism’. We review the theory here.

The theoretical concepts of ‘set points’ and ‘individual euthyroidism’ have been thought to be supported by the finding that the intra-individual variation in TFTs is less than the interindividual variation (1, 8). But though in the presence of a set point, the finding of a lesser intra-individual than inter-individual range implies that the usual values of an individual cannot reliably be predicted from a population range (89), the converse does not necessarily apply, i.e., the presence of lesser intra- than inter-individual variation and the inability to predict the usual values of an individual by reference to the population range do not imply the presence of a set point. The finding of a lesser individual range of variation as compared with the population range is seen with other parameters not believed to be subject to set point physiology (90–93). Furthermore, study of the population FT4/TSH distribution has provided evidence against the existence of thyroid hormone set points (42). Similar studies of other parameters have likewise indicated that set points might not exist at all (94, 95). The stability of any parameters, including individuals’ thyroid hormone levels merely indicates stable equilibrium points which are not necessarily set points (42, 96).

It is true that with primary changes to thyroid hormone levels there are greater changes to the associated TSH levels, i.e., TSH levels are more sensitive to a primary change in thyroid function than are thyroid hormone levels (11, 12). However, the combination of this relationship with the flawed concept that each individual has a set point for thyroid hormones so as to conclude that TSH levels are sensitive indicators of any perturbation from the set point and thereby sensitive indicators of a dysthyroid state (12) only perpetuates an error.

The sensitivity of TSH levels to a primary change in thyroid hormones does not necessarily enable the detection of a dysthyroid state. The evidence against the presence of a set point, and the distribution of pathology within the normal population range imply that it may be for example that an older individual with longstanding, stable, high normal thyroid hormone levels becomes ‘more euthyroid’ if levels drop into the middle or even lower part of the population range, even if this change is associated with a substantial rise in the TSH levels.

And again, this relationship of a parameter having a more sensitive controlling hormone is seen with other parameters e.g., haemoglobin and calcium (97, 98), but this provides no rationale to prefer the use of erythropoietin or parathyroid hormone levels for the assessment as to the presence of anaemia or hypercalcemia. Controlling hormone levels are generally used to determine the cause of any abnormality rather than to detect/define the abnormality (99).

There are in fact physiological reasons why TSH levels might not be congruent with the thyroid state of peripheral tissues. There are differences in deiodinase (87) and ubiquitinase activities (82) centrally and peripherally affecting individual cell response to ambient levels of thyroid hormones in the untreated and treated (82) state. The TSH response is also a function of thyrotrope number (100) whereas the response of peripheral tissues is generally quantified by methods correcting for or independent of cell number (101, 102).

In summary, as well as being inconsistent with empiric data, the theoretical rationale used to support the use of TSH levels for the assessment of the thyroid state may be internally flawed.

Methods of assessing euthyroidism on the basis of paired FT4 and TSH levels have been proposed (3, 103). This move against the emphasis on TSH levels alone, pre-dated the work indicating the superior associations of FT4 levels, as compared with TSH levels, with clinical parameters. The underlying rationale has been based on physiological principles of the appropriate secretion of TSH, leading to the description of the physiological response curves of the FT4/TSH feedback loop, the thyroid and pituitary response curves. The subsequent conclusion was to accept as euthyroid any pair of FT4/TSH values that lies within the normal ranges of the response curves. However, it does not necessarily follow that thyroid and pituitary response curves that in themselves are normal will necessarily generate euthyroid values of thyroid hormones- i.e., a dysthyroid level of FT4 may be generated by a combination of factors neither of which in isolation is definitely abnormal. Furthermore, there are no empiric data supporting this definition of euthyroidism. It has been suggested (3) that individual euthyroidism defined by this method, i.e., ‘the two-dimensional location of the individual equilibrium point (setpoint)….would be the ideal target for dosing algorithms,…’ for replacement therapy. But as previously discussed the evidence indicates that there are no set points for thyroid function (unless set points are defined identically to balance points), and that there is no reason to regard individuals’ usual values of TFTs as particularly ideal or as representing a guide to replacement therapy.

T3 is the active intracellular hormone, and it has been suggested that physiological processes act to maintain stable plasma T3 levels (6). This phenomenon may lead to a diminished sensitivity of T3 levels for the diagnosis of mild hypothyroidism (5). Nevertheless in the meta-analysis of associations with clinical parameters T3/FT3 levels were indeed superior to TSH levels and in a crude analysis were at least as good as FT4 levels (38). In more sophisticated analyses the associations with FT4 levels were more robust. Animal data do indicate that because of local factors affecting thyroid hormone action different tissues may have different intracellular concentrations of T3 and thereby differ in terms of “tissue-specific thyroid status”. Therefore, though FT4 levels appear to be the better marker for euthyroidism this may not apply for all tissues and further studies are indicated (104).

There has been a long interest in T3 replacement therapy complementing T4 replacement. Thus far studies have generally been negative (105), but this has not completely quelled interest (104). Paradoxically the goal of studying outcomes of individuals with different forms of thyroid replacement therapy, particularly combinations of T4 and T3 but with similar TSH level goals (82) implicitly pre-supposes that TSH levels alone are not the best guide for replacement therapy.

## Limitations and Qualifications

The above sections have described changes and recommendation that are general. They do not cover all individuals. We describe here some important qualifications and limitations.

Clinical assessment remains an important part of assessment of the thyroid state. Non-thyroidal illness affecting thyroid function is common (87). Otherwise, as well as determining other pathophysiological states that might influence diagnosis and treatment, it remains important to check that the clinical state is congruent with the biochemical tests. In the context of laboratory artifacts, the assessment of the thyroid state needs to be individualised with co-operation between the clinician and laboratory (106). Thyroid hormone resistance (107) as well as laboratory artifact (106) may be misdiagnosed if reliance is only placed on biochemical evaluation.

There may be differences in clinical parameters depending on whether hypothyroidism is primary or secondary. Some clinical parameters may be affected by TSH levels directly rather than by the associated thyroid state (108, 109)

This review describes the changes to the general architecture of thyroid physiology and the concepts of and measurement of, euthyroidism, thyroid dysfunction and replacement therapy. Such changes to the understanding of the basics of a system do not invalidate true insights obtained under previous conceptions (110). For example, in terms of thyroid physiology and medicine, the validity of previously described details of the peripheral regulation of thyroid hormone physiology such as receptor and de-iodination physiology and action (87) remains unaffected. Such peripheral tissue physiology might however be re-interpreted to lend support to the thesis of this review in that it demonstrates the evolution of physiological processes that might mitigate any adverse effects of any balance point levels of thyroid hormones on organ function.

## Summary and Conclusions

Recent data has enabled the development of a view of euthyroidism and its biomarkers which as well as being consistent with this data, is more consistent than previous paradigms with general physiological principles. **Table 1** provides a summary of these changes in terms of some of the concepts and parameters of thyroid function. In turn these changes have relevance for the successful clinical management of hypothyroidism.

**Table 1:** Summary of revisions proposed for concepts and parameters related to euthyroidism.

Concept/parameter	Traditional view	Revised view
Euthyroidism	State of exposure of all tissues and organs to levels of thyroid hormones optimal for the individual	State of exposure of all tissues and organs to levels of thyroid hormones allowing normal range function, and associated with relatively modest risk profile – levels likely to be in the middle of the population range
Subclinical hypothyroidism (Raised TSH, normal FT4 levels)	State of borderline low thyroid function/mild hypothyroidism, increased risk of progression to overt hypothyroidism.	Spectrum of peripheral thyroid status from euthyroid to borderline low thyroid function. Indicates low functioning thyroid gland and risk of disease progression.
Borderline low thyroid function/mild hypothyroidism	Subclinical hypothyroidism	FT4 levels around the lower limits of the normal range
Healthy individuals’ usual levels of thyroid hormones	Individual euthyroidism/set point largely directly determined genetically	Not necessarily optimal, stable balance point determined by genetically influenced stable feedback loops.
Targets for monitoring thyroid replacement	Normal range TSH levels, adjusted individually	Requires further study. Likely to be levels of FT4 +/- FT3 depending on individual factors and form of replacement therapy
TSH	Most sensitive guide to peripheral tissues thyroid status and preferred parameter for guiding replacement treatment	Inferior guide to peripheral tissues thyroid status. Differentiates primary from secondary hypothyroidism. Prognostic indicator.
FT4	Guide to thyroid status after initial classification according to TSH levels.	The most sensitive and robust measure of the thyroid status of peripheral tissues in untreated individuals. Role in monitoring replacement treatment in primary hypothyroidism requires study.
FT3	Circulating form of the active intracellular hormone. Homeostatically regulated, sensitive to non-thyroidal illness.	Sensitive measure of the peripheral tissue thyroid status. Not as robust as FT4. Requires further study.

Euthyroidism can no longer be thought of as a particular level of thyroid activity, specific for each individual, associated with optimum physiological functioning across all organ systems. All levels, including individuals’ usual levels, of thyroid hormones present different risk profiles which change in a continuous manner, some increasing whilst others are decreasing, with changing hormone levels. Mid-range levels of thyroid hormones, and thereby TSH, might be considered to be ‘most euthyroid’ as such levels are likely to be unassociated with symptoms related to the thyroid state of peripheral tissues, and the risks of a variety of clinical outcomes increase as thyroid hormones diverge from such levels.

Though TSH levels are markers of the thyroid state of peripheral tissues and can help guide thyroid replacement therapy, the traditional teaching that TSH levels are the best biomarkers for these functions and of the state of euthyroidism can no longer be sustained. There is no empiric evidence for such a proposition and the theoretical underpinning of such a position seems to be flawed. There is, on the contrary, strong evidence that levels of thyroid hormones, and in particular of FT4, correlate better with clinical parameters and thereby with euthyroidism. If a single biomarker is to be used for the assessment of the thyroid state the evidence indicates that FT4 levels may be the most accurate.

It seems unlikely that successful treatment of patients can be defined by the achievement of any particular state of psychological well-being without rendering patients subject to other impairments and risks.

Successful treatment of patients with hypothyroidism requires accurate diagnosis and accurate monitoring of replacement therapy. For the more subtle degrees of hypothyroidism there is sufficient evidence to prefer thyroid hormone levels and in particular FT4 levels over TSH levels for the diagnosis and selection of treatment candidates. There is sufficient evidence to no longer consider ‘subclinical hypothyroidism’ as currently defined as an accurate indicator of hypothyroidism of peripheral tissues.

Precise replacement therapy aiming for the restoration of individual euthyroidism is not realistic. For the monitoring of replacement therapy, the evidence suggests that monitoring FT4 and/or FT3 levels, depending on the replacement regimen, may be superior to monitoring TSH levels. There are rational grounds to suggest optimal replacement therapy may result in thyroid hormone levels equivalent in activity to midrange levels in untreated individuals, personalised by consideration of individual preferences and other pathophysiological states. Such monitoring would be somewhat similar to that currently recommended for levothyroxine therapy in secondary hypothyroidism. In these circumstances the proof of successful therapy for any particular individual will remain elusive as clinical outcomes will remain affected by chance. Only group outcomes can be tested. This situation applies to much of medical practice.

The above treatment targets, though rational and consistent with the available evidence, remain unconfirmed by clinical trials and there are therefore opportunities and needs to perform the appropriate studies.

## Author Contributions

SF drafted the paper. HF substantially revised it. Both authors read and approved the final manuscript. Both authors accept accountability for the paper as per the journal’s guidelines.

## Conflict of Interest

The authors declare that the research was conducted in the absence of any commercial or financial relationships that could be construed as a potential conflict of interest.

## Publisher’s Note

All claims expressed in this article are solely those of the authors and do not necessarily represent those of their affiliated organizations, or those of the publisher, the editors and the reviewers. Any product that may be evaluated in this article, or claim that may be made by its manufacturer, is not guaranteed or endorsed by the publisher.
